# 3,4-Di­fluoro-2-hy­droxy­benzoic acid

**DOI:** 10.1107/S1600536814007211

**Published:** 2014-04-05

**Authors:** Bhaskarachar Ravi Kiran, Bandrehalli Siddagangaiah Palakshamurthy, Giriyapura R. Vijayakumar, Hebbur Shivamurthy Bharath

**Affiliations:** a Department of Chemistry, U.C.S., Tumkur University, Tumkur, Karnataka 572 103, India; b Department of Studies and Research in Physics, U.C.S., Tumkur University, Tumkur, Karnataka 572 103, India; c Department of Chemistry, G.F.G.C., Tumkur, Karnataka, 572 102, India

## Abstract

In the title compound, C_7_H_4_F_2_O_3_, an intra­molecular O—H⋯O hydrogen bond is observed. In the crystal, inversion dimers linked by pairs of O—H⋯O hydrogen bonds generate *R*_2_^2^(8) ring motifs. These dimers are linked by C—H⋯O and C—H⋯F hydrogen bonds, forming sheets lying parallel to (30-1). The sheets are linked by aromatic π–π stacking inter­actions [inter-centroid distance = 3.7817 (9) Å], forming a three-dimensional structure.

## Related literature   

For anti­body and gene-directed enzyme prodrug therapy, see: Springer *et al.* (1994 ▶); Davies *et al.* (2005 ▶). For the anti­microbial activity of fluorinated benzoic acid derivatives, see: Rajasekhar *et al.* (2013 ▶).
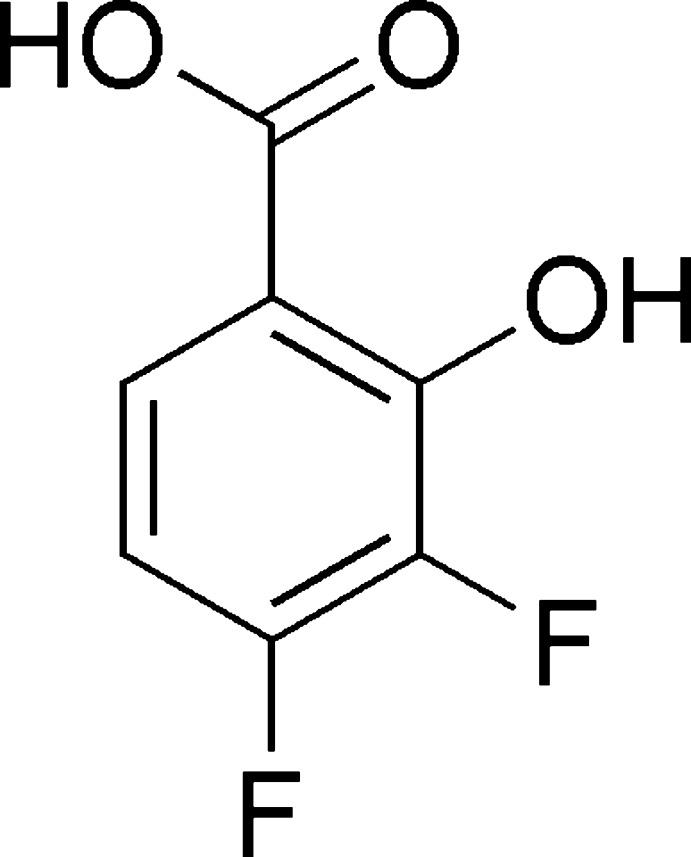


## Experimental   

### 

#### Crystal data  

C_7_H_4_F_2_O_3_
*M* *_r_* = 174.10
Monoclinic, 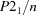

*a* = 9.4252 (8) Å
*b* = 6.8145 (5) Å
*c* = 11.0391 (8) Å
β = 106.257 (5)°
*V* = 680.67 (9) Å^3^
*Z* = 4
Mo *K*α radiation
μ = 0.17 mm^−1^
*T* = 296 K
0.20 × 0.16 × 0.12 mm

#### Data collection  

Bruker APEXII CCD area-detector diffractometer
Absorption correction: multi-scan (*SADABS*; Sheldrick, 2007 ▶) *T*_min_ = 0.967, *T*_max_ = 0.980
6362 measured reflections
1344 independent reflections
1045 reflections with *I* > 2σ(*I*)
*R*_int_ = 0.037

#### Refinement  

*R*[*F* ^2^ > 2σ(*F* ^2^)] = 0.031
*wR*(*F* ^2^) = 0.094
*S* = 1.09
1344 reflections
112 parameters
H-atom parameters constrained
Δρ_max_ = 0.19 e Å^−3^
Δρ_min_ = −0.14 e Å^−3^

### 

Data collection: *APEX2* (Bruker, 2009 ▶); cell refinement: *APEX2* and *SAINT-Plus* (Bruker, 2009 ▶); data reduction: *SAINT-Plus* and *XPREP* (Bruker, 2009 ▶); program(s) used to solve structure: *SHELXS97* (Sheldrick, 2008 ▶); program(s) used to refine structure: *SHELXL97* (Sheldrick, 2008 ▶); molecular graphics: *ORTEP-3 for Windows* (Farrugia, 2012 ▶) and *Mercury* (Macrae *et al.*, 2008 ▶); software used to prepare material for publication: *SHELXL97*.

## Supplementary Material

Crystal structure: contains datablock(s) I, New_Global_Publ_Block. DOI: 10.1107/S1600536814007211/jj2185sup1.cif

Structure factors: contains datablock(s) I. DOI: 10.1107/S1600536814007211/jj2185Isup2.hkl

Supporting information file. DOI: 10.1107/S1600536814007211/jj2185Isup3.cml

CCDC reference: 994805

Additional supporting information:  crystallographic information; 3D view; checkCIF report

## Figures and Tables

**Table 1:** Hydrogen-bond geometry (Å, °)

*D*—H⋯*A*	*D*—H	H⋯*A*	*D*⋯*A*	*D*—H⋯*A*
O3—H3*A*⋯O1	0.82	1.92	2.6231 (14)	144
O2—H2⋯O1^i^	0.82	1.85	2.6679 (14)	175
C3—H3⋯O3^ii^	0.93	2.60	3.5269 (16)	177
C4—H4⋯F2^ii^	0.93	2.53	3.2047 (16)	129

Symmetry codes: (i) 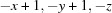; (ii) 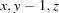.

**Fig. 1.**
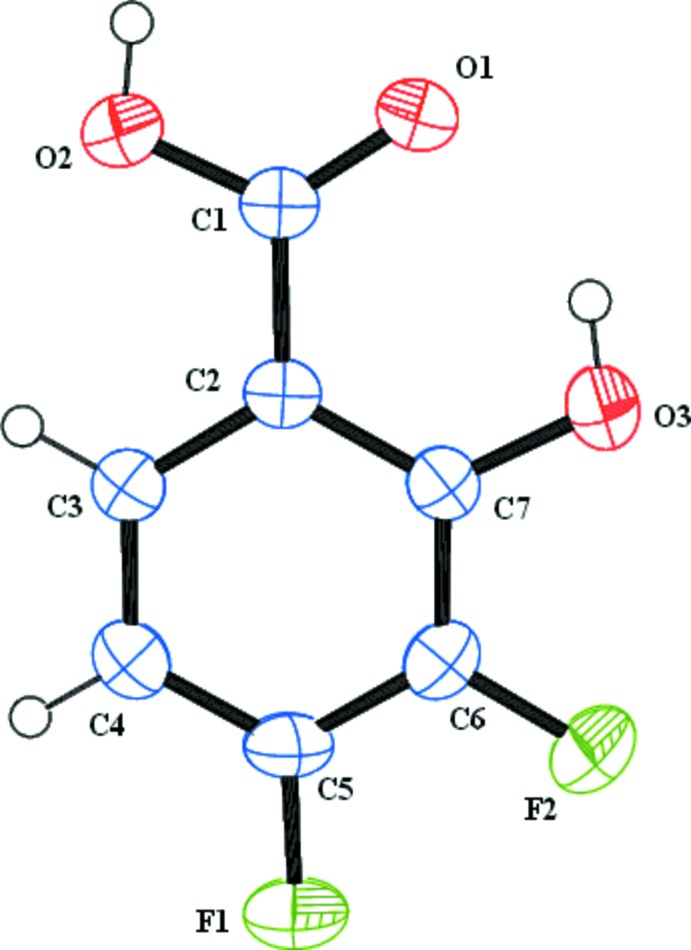
Molecular structure of the title compound, showing displacement ellipsoids drawn at the 50% probability level.

**Fig. 2.**
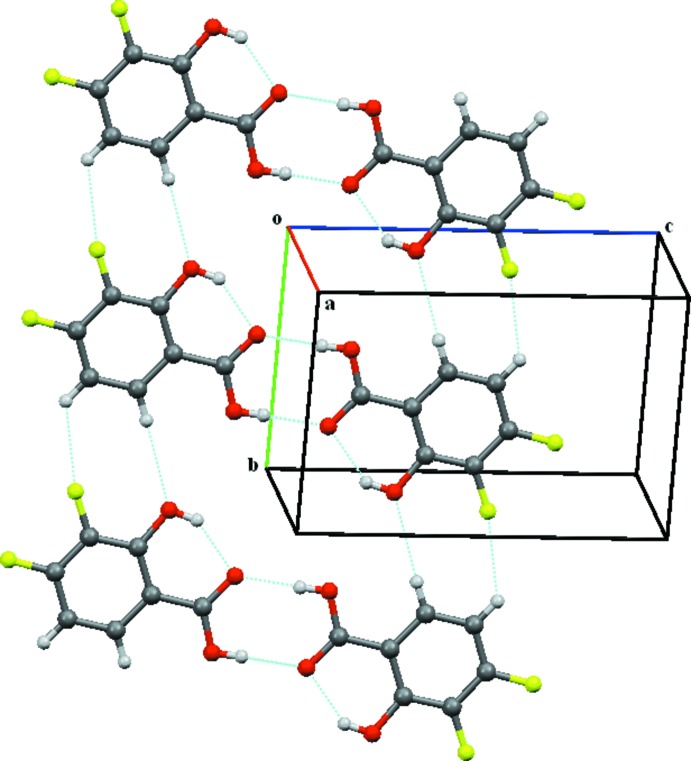
Molecular packing of the title compound viewed along the b axis. Dashed lines indicate O—H···O intramolecular and pairs of O—H···O intermolecular hydrogen bonds forming R22(8) ring motifs and weak C—H···O and C—H···F intermolecular interactions along [010].

**Fig. 3.**
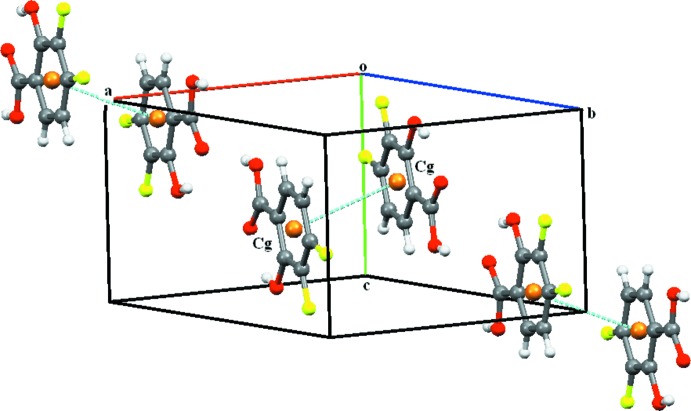
Molecules displaying weak π-π interactions [centroid-centroid separation = 3.7817 (9) Å].

## supplementary crystallographic information

### 1. Comment

Fluorinated benzoic acids have been used for the preparation of potential prodrugs intended for antibody and gene directed enzyme prodrugtherapy (Springer *et al.*, 1994; Davies *et al.*, 2005). Derivatives of fluorinated benzoic acid exhibit antimicrobial activity (Rajasekhar *et al.*, 2013). In particular 3,4-difluoro-2-hydroxybenzoic acid has been used in the synthesis of benzisoxazole containing barbiturate derivatives, which shows prominent anticancer activity (our unpublished results). Hence, the crystal structure of the title compound, (I), C_7_H_4_F_2_O_3,_ is determined.

In (I), the molecule is planar (r.m.s. deviation in the benzene ring = 0.006 (1)Å with a maximum deviation of 0.009 (1)Å for carbon) (Fig. 1). An intramolecular O3—H3A···O1 hydrogen bond in observed. In the crystal, inversion dimers linked by pairs of O2—H2···O1 hydrogen bonds are formed and generate *R*_2_^2^(8) ring motifs (Fig. 2). Weak C3—H3···O3 and C4—H4···F2 intermolecular interactions and aromatic π-π stacking interactions [centroid-centroid separation = 3.7817 (9) Å] (Fig. 3) are also observed and contribute to packing stability.

### 2. Experimental

To an ice cooled and stirred solution of 2,3,4-trifluorobenzoic acid (0.028 mmol) in dimethylimidazolidinone (10 ml), solid sodium hydroxide (0.113 mmol) was added in portions, and the mixture was heated to 120°C for 2 h. The reaction was monitored by TLC. After the reaction was completed, the mixture was cooled to room temperature and neutralized (pH 5–6) with 2 N hydrochloric acid (7.5 ml). The title compound was separated out as white solid, filtered, washed with excess of water and dried. Colourless prisms of the title compound were grown in ethanol by slow the evaporation technique.

### 3. Refinement

The hydroxy H-atoms were located in a difference Fourier map, and were refined isotropically with the O–H distance restrained to 0.82±0.01 Å. H atoms were positioned with idealized geometry using a riding model with C—H = 0.93 Å and were refined with isotropic displacement parameters (set to 1.2 times of the *U*_eq_ of the parent atom).

### Crystal data

**Table:**

C_7_H_4_F_2_O_3_	Prism
*M**_r_* = 174.10	*D*_x_ = 1.699 Mg m^−^^3^
Monoclinic, *P*2_1_/*n*	Melting point: 448 K
Hall symbol: -P 2yn	Mo *K*α radiation, λ = 0.71073 Å
*a* = 9.4252 (8) Å	Cell parameters from 1045 reflections
*b* = 6.8145 (5) Å	θ = 2.3–26.5°
*c* = 11.0391 (8) Å	µ = 0.17 mm^−^^1^
β = 106.257 (5)°	*T* = 296 K
*V* = 680.67 (9) Å^3^	Prism, colourless
*Z* = 4	0.20 × 0.16 × 0.12 mm
*F*(000) = 352	

### Data collection

**Table:**

Bruker APEXII CCD area-detector diffractometer	1344 independent reflections
Radiation source: fine-focus sealed tube	1045 reflections with *I* > 2σ(*I*)
Graphite monochromator	*R*_int_ = 0.037
Detector resolution: 1.6 pixels mm^-1^	θ_max_ = 26.0°, θ_min_ = 2.5°
phi and ω scans	*h* = −11→11
Absorption correction: multi-scan (*SADABS*; Sheldrick, 2007)	*k* = −8→8
*T*_min_ = 0.967, *T*_max_ = 0.980	*l* = −13→13
6362 measured reflections	

### Refinement

**Table:**

Refinement on *F*^2^	Secondary atom site location: difference Fourier map
Least-squares matrix: full	Hydrogen site location: inferred from neighbouring sites
*R*[*F*^2^ > 2σ(*F*^2^)] = 0.031	H-atom parameters constrained
*wR*(*F*^2^) = 0.094	*w* = 1/[σ^2^(*F*_o_^2^) + (0.0514*P*)^2^ + 0.0514*P*] where *P* = (*F*_o_^2^ + 2*F*_c_^2^)/3
*S* = 1.09	(Δ/σ)_max_ < 0.001
1344 reflections	Δρ_max_ = 0.19 e Å^−^^3^
112 parameters	Δρ_min_ = −0.14 e Å^−^^3^
0 restraints	Extinction correction: *SHELXL97* (Sheldrick, 2008), Fc^*^=kFc[1+0.001xFc^2^λ^3^/sin(2θ)]^-1/4^
0 constraints	Extinction coefficient: 0.018 (3)
Primary atom site location: structure-invariant direct methods	

### Special details

**Table:**

Geometry. All esds (except the esd in the dihedral angle between two l.s. planes) are estimated using the full covariance matrix. The cell esds are taken into account individually in the estimation of esds in distances, angles and torsion angles; correlations between esds in cell parameters are only used when they are defined by crystal symmetry. An approximate (isotropic) treatment of cell esds is used for estimating esds involving l.s. planes.
Refinement. Refinement of F^2^ against ALL reflections. The weighted R-factor wR and goodness of fit S are based on F^2^, conventional R-factors R are based on F, with F set to zero for negative F^2^. The threshold expression of F^2^ > 2sigma(F^2^) is used only for calculating R-factors(gt) etc. and is not relevant to the choice of reflections for refinement. R-factors based on F^2^ are statistically about twice as large as those based on F, and R- factors based on ALL data will be even larger.

### Fractional atomic coordinates and isotropic or equivalent isotropic displacement parameters (Å^2^)

**Table:**

	*x*	*y*	*z*	*U*_iso_*/*U*_eq_	
C1	0.55955 (13)	0.54118 (17)	0.18180 (14)	0.0376 (3)	
C2	0.60924 (12)	0.57452 (17)	0.31759 (13)	0.0357 (3)	
C3	0.64443 (13)	0.41737 (18)	0.40234 (13)	0.0400 (3)	
H3	0.6343	0.2897	0.3713	0.046 (4)*	
C4	0.69337 (14)	0.44745 (19)	0.52991 (15)	0.0458 (4)	
H4	0.7180	0.3420	0.5854	0.065 (5)*	
C5	0.70534 (14)	0.63748 (19)	0.57424 (14)	0.0443 (3)	
C6	0.67106 (15)	0.79441 (18)	0.49292 (15)	0.0441 (4)	
C7	0.62456 (13)	0.76688 (16)	0.36434 (14)	0.0380 (3)	
O1	0.53519 (11)	0.67798 (13)	0.10424 (9)	0.0482 (3)	
O2	0.54221 (10)	0.35741 (12)	0.14643 (10)	0.0503 (3)	
H2	0.5139	0.3516	0.0692	0.075*	
O3	0.59795 (11)	0.92951 (13)	0.29161 (10)	0.0543 (3)	
H3A	0.5708	0.8976	0.2170	0.081*	
F1	0.75075 (11)	0.67350 (13)	0.69839 (8)	0.0676 (3)	
F2	0.68666 (12)	0.97767 (11)	0.54053 (10)	0.0686 (3)	

### Atomic displacement parameters (Å^2^)

**Table:**

	*U*^11^	*U*^22^	*U*^33^	*U*^12^	*U*^13^	*U*^23^
C1	0.0438 (6)	0.0373 (7)	0.0310 (9)	−0.0007 (5)	0.0095 (6)	−0.0010 (5)
C2	0.0417 (6)	0.0361 (7)	0.0292 (9)	−0.0008 (4)	0.0096 (6)	−0.0007 (5)
C3	0.0536 (7)	0.0331 (6)	0.0325 (9)	0.0016 (5)	0.0107 (6)	0.0007 (5)
C4	0.0604 (8)	0.0391 (7)	0.0356 (10)	0.0030 (5)	0.0095 (7)	0.0064 (5)
C5	0.0544 (7)	0.0511 (8)	0.0248 (9)	−0.0034 (5)	0.0065 (6)	−0.0039 (6)
C6	0.0569 (7)	0.0347 (7)	0.0395 (10)	−0.0068 (5)	0.0113 (7)	−0.0071 (5)
C7	0.0470 (7)	0.0337 (6)	0.0323 (10)	−0.0032 (5)	0.0097 (6)	0.0017 (5)
O1	0.0712 (6)	0.0401 (5)	0.0296 (7)	−0.0009 (4)	0.0077 (5)	0.0017 (4)
O2	0.0771 (6)	0.0378 (5)	0.0319 (7)	−0.0016 (4)	0.0087 (5)	−0.0040 (4)
O3	0.0836 (7)	0.0340 (5)	0.0402 (7)	−0.0031 (4)	0.0091 (6)	0.0042 (4)
F1	0.0996 (7)	0.0677 (6)	0.0287 (6)	−0.0032 (5)	0.0065 (5)	−0.0070 (4)
F2	0.1113 (7)	0.0403 (5)	0.0474 (7)	−0.0096 (4)	0.0111 (6)	−0.0140 (4)

### Geometric parameters (Å, º)

**Table:**

C1—O1	1.2429 (15)	C4—H4	0.9300
C1—O1	1.2429 (15)	C5—F1	1.3392 (16)
C1—O2	1.3083 (14)	C5—C6	1.375 (2)
C1—C2	1.458 (2)	C6—F2	1.3469 (14)
C2—C3	1.3994 (18)	C6—C7	1.376 (2)
C2—C7	1.4014 (17)	C7—O3	1.3502 (16)
C3—C4	1.369 (2)	O2—H2	0.8200
C3—H3	0.9300	O3—H3A	0.8200
C4—C5	1.3777 (19)		
			
O1—C1—O2	121.92 (13)	C5—C4—H4	120.8
O1—C1—O2	121.92 (13)	F1—C5—C6	118.35 (12)
O1—C1—C2	122.39 (11)	F1—C5—C4	120.45 (12)
O1—C1—C2	122.39 (11)	C6—C5—C4	121.21 (14)
O2—C1—C2	115.69 (11)	F2—C6—C5	119.09 (14)
C3—C2—C7	119.27 (13)	F2—C6—C7	119.82 (12)
C3—C2—C1	121.06 (11)	C5—C6—C7	121.06 (12)
C7—C2—C1	119.66 (11)	O3—C7—C6	117.00 (12)
C4—C3—C2	121.45 (12)	O3—C7—C2	124.47 (14)
C4—C3—H3	119.3	C6—C7—C2	118.53 (12)
C2—C3—H3	119.3	C1—O2—H2	109.5
C3—C4—C5	118.46 (13)	C7—O3—H3A	109.5
C3—C4—H4	120.8		
			
O1—C1—C2—C3	−176.05 (11)	F1—C5—C6—C7	179.65 (11)
O1—C1—C2—C3	−176.05 (11)	C4—C5—C6—C7	−0.5 (2)
O2—C1—C2—C3	3.89 (17)	F2—C6—C7—O3	0.61 (19)
O1—C1—C2—C7	2.86 (17)	C5—C6—C7—O3	−177.90 (11)
O1—C1—C2—C7	2.86 (17)	F2—C6—C7—C2	−179.95 (10)
O2—C1—C2—C7	−177.19 (10)	C5—C6—C7—C2	1.5 (2)
C7—C2—C3—C4	0.05 (18)	C3—C2—C7—O3	178.06 (11)
C1—C2—C3—C4	178.97 (10)	C1—C2—C7—O3	−0.87 (18)
C2—C3—C4—C5	1.03 (19)	C3—C2—C7—C6	−1.33 (18)
C3—C4—C5—F1	179.05 (11)	C1—C2—C7—C6	179.74 (10)
C3—C4—C5—C6	−0.8 (2)	O2—C1—O1—O1	0.00 (14)
F1—C5—C6—F2	1.1 (2)	C2—C1—O1—O1	0.00 (14)
C4—C5—C6—F2	−178.98 (12)		

### Hydrogen-bond geometry (Å, º)

**Table:**

*D*—H···*A*	*D*—H	H···*A*	*D*···*A*	*D*—H···*A*
O3—H3*A*···O1	0.82	1.92	2.6231 (14)	144
O2—H2···O1^i^	0.82	1.85	2.6679 (14)	175
C3—H3···O3^ii^	0.93	2.60	3.5269 (16)	177
C4—H4···F2^ii^	0.93	2.53	3.2047 (16)	129

Symmetry codes: (i) −*x*+1, −*y*+1, −*z*; (ii) *x*, *y*−1, *z*.
